# Response: Commentary: Immunogenic Cell Death and Immunotherapy of Multiple Myeloma

**DOI:** 10.3389/fcell.2019.00306

**Published:** 2019-11-27

**Authors:** Alfonso Serrano Del Valle, Alberto Anel, Javier Naval, Isabel Marzo

**Affiliations:** Biochemistry and Molecular and Cell Biology, University of Zaragoza, Zaragoza, Spain

**Keywords:** immunogenic cell death, multiple myeloma, ER stress, danger-associated molecular pattern, immunotherapy

“Immunotherapy of myeloma by using immunogenic dead cells seems to be more complex than anticipated.”

We have read with great interest and appreciated the Commentary of Ken Maes and Karine Breckpot pointing out the difficulties to achieve an efficient vaccination using myeloma cells killed by using anti-tumor drugs, in such a way to generate immunogenic corpses able to elicit protective immunity in patients. In this sense, the pioneer studies of De Beck et al. (2018), clearly illustrated the problems to achieve an immunization protocol providing a 100% protection. Although complete protection from myeloma development could not be reached, vaccination with drug-treated myeloma cells induced a delay in tumor progression (De Beck et al., 2018). This relevant work was inadvertently omitted in our previous review on immunogenic cell death (ICD) and immunotherapy of multiple myeloma (Serrano-Del Valle et al., 2019). Moreover, in experiments carried out after the publication of our review, we could not prevent induced myeloma in an immunocompetent mouse model that mimics human myeloma development, by vaccination with apoptotic myeloma cells killed with bona fide ICD inducers, which cause ecto-calreticulin exposure (data not shown). These results also illustrate the inability of current immunization protocols to completely prevent myeloma development, underscoring the problems discussed by Maes and Breckpot (2019). We also agree with these authors on which the ICD-inducing capacities of dying MM cells needs to be better defined to design more efficient, improved immunization protocols in immunocompetent mice as a first step to translate this information to human myeloma therapy.

## Author Contributions

All authors listed have made a substantial, direct and intellectual contribution to the work, and approved it for publication.

### Conflict of Interest

The authors declare that the research was conducted in the absence of any commercial or financial relationships that could be construed as a potential conflict of interest.
